# Addressing Multi-User Interference in Vehicular Visible Light Communications: A Brief Survey and an Evaluation of Optical CDMA MAC Utilization in a Multi-Lane Scenario

**DOI:** 10.3390/s23083831

**Published:** 2023-04-08

**Authors:** Emmanuel Plascencia, Hongyu Guan, Luc Chassagne, Alin-Mihai Căilean, Olivier Barrois, Oyunchimeg Shagdar

**Affiliations:** 1 Oledcomm, 78140 Vélizy, France; 2 Systems Engineering Laboratory of Versailles, University of Versailles Saint-Quentin-en-Yvelines, University of Paris-Saclay, 78140 Vélizy, France; 3 Department of Computers, Electronics and Automation, Stefan Cel Mare University of Suceava, 720229 Suceava, Romania; 4 Integrated Center for Research, Development and Innovation in Advanced Materials, Nanotechnologies and Distributed Systems for Fabrication and Control, Stefan Cel Mare University of Suceava, 720229 Suceava, Romania; 5 Renault Group, 78084 Guyancourt, France

**Keywords:** multi-user interference, multi-user interference mitigation, OCDMA, optical interference, vehicle-to-vehicle communications, V2V, VLC, VLC MAC solutions, visible light communications

## Abstract

Visible Light Communications (VLC) are developing as an omnipresent solution for inter-vehicle communications. Based on intensive research efforts, the performance of vehicular VLC systems has significantly improved in terms of noise resilience, communication range, and latencies. Nevertheless, in order to be ready for deployment in real applications, solutions for Medium Access Control (MAC) are also required. In this context, this article provides an intensive evaluation of several optical CDMA MAC solutions and of their efficiency in mitigating the effect of Multiple User Interference (MUI). Intensive simulation results showed that an adequately designed MAC layer can significantly reduce the effects of MUI, ensuring an adequate Packet Delivery Ratio (PDR). The simulation results showed that based on the use of optical CDMA codes, the PDR can be improved from values as low as 20% up to values between 93.2% and 100%. Consequently, the results provided in this article show the high potential of optical CDMA MAC solutions in vehicular VLC applications, reconfirm the high potential of the VLC technology in inter-vehicle communications, and emphasize the need to further develop MAC solutions designed for such applications.

## 1. Introduction

In the current intelligent and autonomous vehicle paradigm, communications have become a new type of intelligent sensor. In this area, wireless communications are envisioned to facilitate traffic Infrastructure-to-Vehicle (I2V) and Vehicle-to-Vehicle (V2V) communications, contributing in turn to improved road safety and efficiency [1,2,3,4]. The automotive illumination technology is a different sector that has seen a substantial evolution in previous years. In this domain, Light Emitting Diodes (LED) lighting sources are close to completely replacing traditional lighting devices [5,6,7,8], whereas the new laser-based lighting systems are prepared as the next step in this domain. Thus, compared to the use of traditional xenon or halogen bulbs, the current and the future generation of Solid State Lighting (SSL) sources provide numerous benefits, emphasizing here the long usable life, the low power consumption, the high environmental tolerance, the high-efficiency, the improved visibility, and the superior adaptability [5,6,7,8]. In this context, Visible Light Communications (VLC) have developed as a viable wireless communication technology that was driven by significant progress in SSL technology and by the widespread use of LEDs in indoor and outdoor lighting applications. Thus, VLC is a novel technique that enables data transmission by altering the intensity of the light generated by SSL devices [8,9,10,11]. Therefore, VLC uses the visible light spectrum (i.e., wavelengths between 380 and 780 nm) for simultaneous illumination and data transmission. In vehicular applications, VLC is enabled due to the integration of SSL devices in vehicle headlights and taillights, and, as a result, its implementation is quick, safe, and cost-efficient [8,11].

In the Intelligent Transportation System (ITS) concept, vehicle safety and transportation efficiency can be provided based on the integration of wireless communications, enabling vehicle and traffic infrastructures to share relevant road safety data. IEEE 802.11p represents the most advanced and general acceptance solution based on its well-confirmed overtime performances [12,13]. Nevertheless, intensive use of 802.11p solutions might generate reliability issues that will affect Packet Delivery Ratio (PDR) and the overall communication latency [14,15,16]. In this context, numerous works have demonstrated the complementarity between VLC and IEEE 802.11p solutions, generating improved performances, resilience to attacks, resilience to interference, improved connectivity, etc. [14,15,16].

In terms of performance, vehicular VLC systems have been significantly improved in recent years. Accordingly, based on intensive research efforts, the most significant challenges from vehicular VLC systems [8] have been partially resolved. Thus, the resilience to noise of such systems has been significantly improved, enabling reliable links even in optical interference conditions [17,18,19] or in unfriendly weather conditions [19,20]. For example, communication links around 50 m have been demonstrated in direct sunlight exposure or in snowfall conditions. The communication range has also significantly increased, reaching today almost 200 m [21,22], while providing 100 kb/s data rates. Moreover, inter-vehicle commutations in situations similar to the real-world scenario have been demonstrated as well [23,24,25,26], including scenarios in which the VLC transmitter and the VLC receiver are misaligned or vehicles that are in highway conditions. Additionally, it has been experimentally demonstrated that vehicular VLC systems are capable of providing very low latencies of only a few ms [19,27].

As shown above, vehicular VLC systems have made great progress in terms of PHY layer development [8,9,10,11,12,13,14,15,16,17,18,19,20,21,22,23,24,25,26,27]. Nevertheless, due to the fact that in the initial development stage such systems were considered as strictly point-to-point links with narrow reception angles, the influence of multi-path effects or of neighboring vehicular VLC links has not been investigated or has been insufficiently addressed. From this point of view, vehicular VLC receivers were considered as having very narrow Field-of-Views (FoV), and, thus, the effects of Multi User Interference (MUI) were mostly neglected. On the other hand, as the performance of vehicular VLC systems have reached a level that is high enough to be used in real applications, the issues associated with MUI become very important, as solving these issues could be one of the last steps in order to establish the viability of VLC use in vehicular applications, contributing in this way towards VLC technology utilization.

In the abovementioned context, this article approaches the issues associated with the mutual interference of inter-vehicle VLC links, providing an up-to-date analysis of existing solutions. In addition, it provides a complex simulation analysis concerning the utilization of Optical Code Division Multiple Access (OCDMA) techniques as a solution to mitigate the effects of MUI in vehicular VLC applications. This work carries on the work begun in [28] by providing a more complex analysis of existing solutions to address MUI in vehicular applications. In addition, different from [28], which investigated the benefits of two OCDMA codes in MUI mitigation, this new article provides additional results and a more comprehensive analysis, focusing on the evaluation of four optical CDMA codes. Thus, this work emphasizes that different sub-codes may have different cross-correlation properties, which, in turn, can lead to variable performances. The other main contributions of this article are represented by a comprehensive analysis of existing solutions designed to mitigate the effects of MUI in vehicular applications, and by a deep analysis and an evaluation of optical CDMA codes as a response to this problem. Therefore, we consider that this work provides encouraging results with respect to the utilization of the VLC technology in V2V applications, as it demonstrates that based on an adequate design of the MAC layer, the effects of MUI can be significantly reduced, and that in such a case, MUI do not significantly affect vehicular VLC PDR performance. In addition, this work provides a comprehensive analysis summarizing the lessons that have been learned in the MUI mitigation domain and indicating some challenges for the future development of MUI mitigation in vehicular VLC applications.

The rest of this work is structured as follows. Section 2 approaches the issues related to multiple user interferences in vehicular VLC applications and provides an overview on the existing state of the art focused on vehicular VLC MAC and on OCDMA code development. Section 3 presents the four codes selected for the evaluation process, whereas Section 4 provides the summary of the simulation results showing the benefits of the four OCDMA codes. Finally, Section 5 provides a discussion concerning the findings of this article and its conclusions.

## 2. Vehicular Visible Light Communications Multi-User Interference Issues and Solutions for Their Mitigation: State of the Art

### 2.1. Debate on the Necesity of a MAC Solution for Vehicular Visible Light Communications Applications

**Figure 1:**
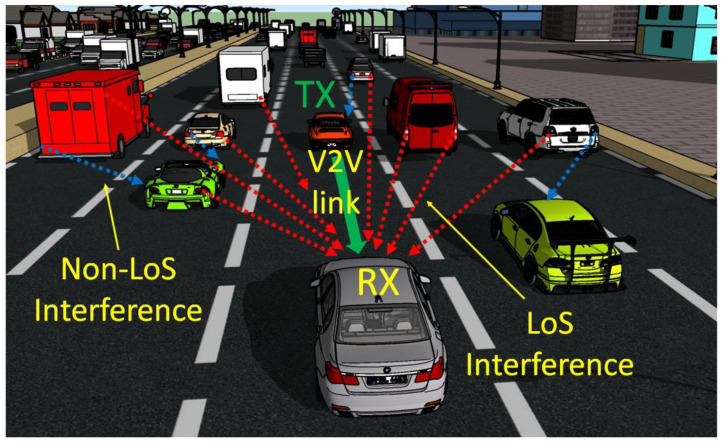
Graphical illustration of the multi-user interference appearance in an urban highway scenario: in addition to the message received from the intended VLC transmitter (TX), the VLC receiver (RX) is also exposed to interferences from other vehicles within its line of sight.

As discussed in Section 1, the VLC technology is struggling to receive its share in vehicular communication applications. In this area, the VLC technology benefits from unique advantages, and it has made impressive progress in a relatively short space of time. According to some research, VLC should be used in conjunction with 5.9 GHz Dedicated Short Range Communications (DSRC) governed by the IEEE 802.11p standard [12,13,14,15,16]. In its turn, the VLC technology has taken advantage of being a new concept and benefited from many of the 5.9 GHz DSRC lessons. Now, if it is to further follow this successful path, one can see that a major step in the development of the DSRC technology consisted in the ability to manage multiple users simultaneously [29,30,31]. Therefore, although in the initial phase it seemed that the high number of nodes and the large amount of interference are a major vulnerability, intensive research efforts increased 5.9 GHz DSRC reliability [29,30,31]. Consequently, in order to improve vehicular VLC reliability, the development of MAC solutions should be addressed as well. Thus, although vehicular VLC applications are less exposed to MUI, this issue could affect performances of such links. An illustration of such a situation is exemplified in Figure 1.

Before developing new MAC solutions for improved vehicular VLC systems, demonstrating their necessity is important. Therefore, in [25], it has been experimentally demonstrated that a typical VLC emitter developed based on a vehicular tail-lamp set has an emission angle up to 60°, enabling signal reception from a neighboring lane for a distance of more than 60 m. In [32], analytical and simulation investigations showed that the effects of MUI on inter-vehicle VLC links are dependent on the V2V separation distance, on the number of lanes, and on the vehicle density. The authors of [33] have evaluated the use of VLC in platooning applications and concluded that the technology is suitable for such uses, but it is strongly affected by MUI, which significantly increases message failure rate. The use of VLC for vehicular networks cellular offloading has been addressed in [34]. Again, simulation results demonstrated the benefits of VLC utilization. On the other hand, a careful analysis of vehicular VLC systems parameters indicated that coverage area is limited by narrow receiver FOV, whereas the FOV increase leads to a higher number of neighboring vehicles within the Line of Sight (LoS), generating in turn a higher collision probability and lower data delivery percentage [34]. An intensive analytical and experimental evaluation demonstrating the highly disruptive effects of MUI on inter-vehicle VLC can be found in [35]. Here [35], computer simulations and experimental investigations showed that depending on the MUI intensity, the PDR can be significantly affected, reaching in certain conditions values below 40%. The authors of [36,37] perform analyses of the vehicular VLC channel model based on the impact of real vehicle lighting system radiation patters. These studies showed that V2V communication coverage and, in turn, VLC MUI area are highly dependent on each vehicle manufacturer, and that in many cases, this coverage can be extensive. The fact that a dedicated MAC is required for vehicular VLC applications is also demonstrated in [38]. The authors argue that in the initial development stage, vehicular VLC applications seem not to be affected by MUI, and, as a result, MAC development has not been considered. Nevertheless, simulation results provided by [38] show that in certain conditions, an on-vehicle VLC receiver can be within the LoS of up to 30 VLC transmitters, taking packet losses up to 13%. The importance of a MAC protocol dedicated to vehicular VLC use is also emphasized in [39], where the authors show that the original IEEE 802.11 MAC protocol is not able to provide the expected results, leading to a 70% PDR and increased latencies. Although focusing on indoor applications, the authors of [40] bring to light another possible phenomena that could generate problems in vehicular VLC as well. In RF-based vehicular communications, the hidden node problem could also affect vehicular VLC applications, especially in the context in which the sensibility and the coverage of VLC receivers are constantly improving.

In conclusion, although the effects of MUI in vehicular VLC are not fully investigated, one can see based on [25,28,32,33,34,35,36,37,38,39,40] that these effects exist and that they do affect PDR, latencies, and overall link reliability. Consequently, solutions to this issue should be further investigated. 

### 2.2. Medium Acces Control Techniques for Visible Light Communications Applications

When discussing MAC techniques, Time Division Multiple Access (TDMA), Frequency Division Multiple Access (FDMA), Carrier Sense Multiple Access with Collision Avoidance (CSMA/CA), Space-Division Multiple Access (SDMA), ALOHA, and CDMA are some of the available options. As the general characteristics of these techniques are rather well known, whereas the issues associated with their use in VLC networks have been addressed in [41,42,43], this work will not provide an additional discussion on their characteristics. As summarized in [28], CSMA/CA and ALOHA are not able to provide collision-free channel access, whereas TDMA is mostly suitable for centralized networks, being rather difficult to implement in decentralized networks, such as in Vehicular Adhoc Networks (VANET). On the other hand, using OFDM seems to be promising as this technique offers the premises for proper management of multiple users, resilience to optical noise sources, and high data rates [44,45]. Nevertheless, although OFDM has high potential, its use requires a complex hardware implementation, and, as a consequence, there are only a few examples of hardware vehicular VLC prototypes reported in the literature.

In turn, CDMA comes as a simple alternative to providing multi-user support in vehicular VLC applications. Therefore, CDMA enables multiple user access based on the spread spectrum technology with vehicles (network nodes) that use a specific spreading code. CDMA codes can be classified as synchronous and asynchronous. Synchronous codes offer robust cross correlation characteristics but require perfect time synchronization. On the other hand, asynchronous spreading codes are less performant in cross correlation, while having the advantage of not being sensitive to node synchronization.

### 2.3. Solutions to Address Multi-User Interference in Vehicular Visible Light Communications Applications

Once the problem has been identified, solutions must be provided. Therefore, it should be stated that the issues associated with multi-user handling and MUI mitigation have been mainly addressed in indoor VLC applications, while being rather neglected in the case of vehicular VLC applications. On the other hand, it is obvious the once the MUI issues are solved for indoor applications, the lessons learned will be adopted and applied in vehicular uses as well.

The authors of [46] propose the Access SDMA protocol, which is joined with an adaptive data rate control as a solution for improved performance MAC in VANETs. The proposed solution improved overall performance and throughput by using SDMA for collision mitigation and adapted packet data rates selected based on vehicle density. Stimulated by the advances of the SSL industry and by the development of adaptive vehicle front-lighting systems, the authors of [47,48] also analyze the use of SDMA as a solution to reduce MUI in vehicular VLC applications. Hence, they consider that VLC should take advantage of new generation vehicle matrix lights. On these grounds, they propose using these improved vehicle lighting solutions to enable SDMA for multi-user medium access in vehicular VLC networks. The authors recommend a location-aware concept, in which matrix lights are used to avoid MUI. Intensive simulation results clearly showed the benefits of such a space-division protocol, the protocol enabling a superior spatial reuse, and a simple and efficient multi-user channel access. Unsatisfied by the performance of SDMA and Non-Orthogonal Multiple Access (NOMA) solutions, the author of [49] propose a rate-splitting multiple access mechanism that seems to provide improved results in terms of achievable weighted sum rate. Although not strictly related to the problem of MUI, the authors of [50] emphasize the importance of edge computing in VANETs, as a solution to improve computational capabilities of in-vehicle embedded systems. In turn, such solutions could be used in better handling the issues generated by MUI based on their superior computational capabilities. A different approach in dealing with MUI in vehicular VLC applications is found in [51]. Here, the authors focus on the use of CDMA to enable multi user access and propose the employment of an inverted Modified Prime Sequence Codes (MPSCs) in optical CDMA VLC systems. As a result, the authors claim that this approach increases brightness, while having a similar BER as non-inverted MPSC. The utilization of optical CDMA for vehicular VLC applications is also investigated in [28]. In [28], PN sequence codes are introduced and compared with Optical Orthogonal Codes (OOCs) codes. The authors conclude that PN sequence OCDMA could be attractive in vehicular VLC applications as they do not require synchronization, simplifying data exchange in real V2V scenarios. A more complex solution is proposed in [52], where a protocol that integrates optical CDMA and TDMA is evaluated in a VANET configuration. Simulation results showed an average access delay of 2.75 ms even in the worst situations, whereas the use of OOC codes significantly improves network throughput. In [53], a CSMA/CA MAC layer is investigated in a vehicular utilization scenario. Simulation results showed that the CSMA/CA protocol can ensure a reliable PDR, while also indicating the vulnerabilities generated by unfriendly weather conditions. Therefore, in the context in which both VLC and 5.9 GHz DSRC solutions have certain reliability issues, the authors of [54], investigate the benefits of heterogeneous VLC-RF networks, and they analyze the benefits of a MAC solution that integrates both technologies. The results show that such a hybrid VLC-RF MAC solution provides substantial improvement in terms of outage performance, throughput, and latency compared to either the stand-alone solutions. Rather similar conclusions are also provided by [55].

In light of the information presented in Section 1 and Section 2, it should be emphasized that most of the current VLC efforts are concentrated on improving physical layer architecture. Additionally, one can see that the recent works clearly suggest that MAC development is required for VLC in automotive applications, whereas current literature contains relatively limited responses on these issues. Thus, one can see that there are relatively few works that have adequately addressed the issues associated with vehicular MAC VLC development, emphasizing that additional research efforts should be oriented toward this direction as well. This concern is also emphasized in [55]. On the other hand, the lack of a MAC design postpones VLC utilization in VANET. Consequently, non-beacon mode without CSMA/CA, non-beacon mode with CSMA/CA, beacon-enabled mode without CSMA/CA, and beacon-enabled mode with CSMA/CA are random multiple access techniques stipulated by the current IEEE 802.15.7 standard [56,57], further emphasizing the importance of MAC development.

## 3. Optical Codes for Visible Light Communications Multiple Access

The work focused on multi-user access in VLC networks has mentioned and investigated several codes that could be used in different setups. Modified Double Weight (MDW) codes are an example of such an option [58]. MDW codes can provide low cross-correlation properties suitable to mitigate the effects of multipath fading and improving the Signal-to-Noise Ratio (SNR). On the downside, these codes generate a complex design and implementation, especially for applications that involve a large number of users. Another example are the Hadamard codes, which are orthogonal codes that have been considered especially in indoor VLC applications [59]. In such applications, these codes are considered suitable to provide high data rates and BER. Nevertheless, their investigation showed some vulnerabilities when these codes are used in the presence of a strong DC component, leading to a higher BER [59]. Therefore, as vehicular VLC applications should work in the presence of strong sunlight, which generates, in turn, a strong DC component, the utilization of Hadamard codes in vehicular VLC applications is for the moment postponed. Additionally, these codes have limited length, limiting the number of users that can be supported. 

Based on the reasons that will be further detailed, four different codes have been selected for further evolution in this work. A specific issue associated with CDMA MAC systems is related to code allocation. In this article, we consider a CDMA MAC protocol that enables nodes to select CDMA codes independently, depending on their location and direction. As a result, this approach no longer requires information sharing between potential interfering nodes. The following section aims to provide a brief description of these four codes, emphasizing their main benefits but also their disadvantages.

### 3.1. Random Optical Codes

**Figure 2:**
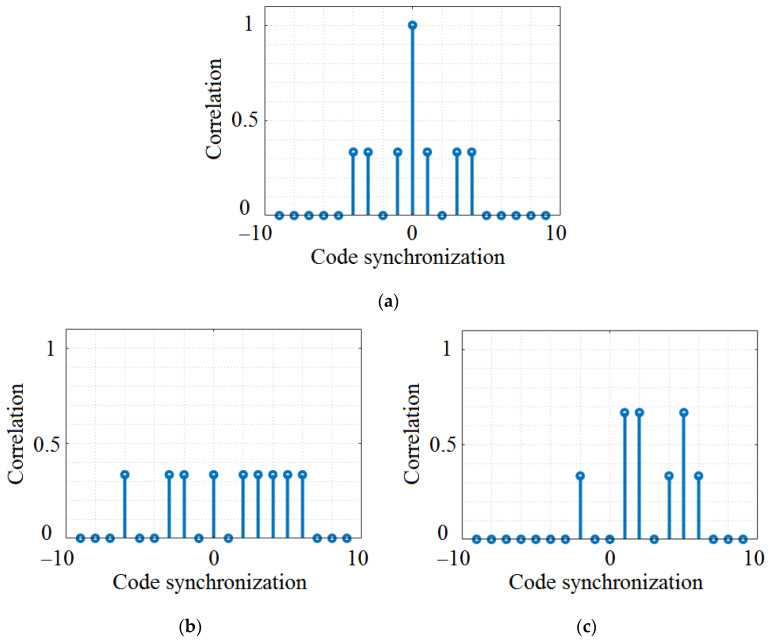
Analysis of ROC correlation properties: (**a**) ROC3 auto-correlation property; (**b**) example of low cross-correlation between ROC3 and ROC4; (**c**) example of high cross-correlation between ROC3 and ROC1.

Random Optical Codes (ROCs) are compatible with applications that assume a relatively high number of users that share the same communication channel. ROCs have been investigated for utilization in vehicular VLC OCDMA scenarios as a substitute to traditional OCDMA codes. ROCs randomly generate unique codes, without necessitating a pre-designed code set. The benefit of ROCs use is that these codes can provide a high degree of flexibility and can be generated quickly and easily. This can be particularly convenient in vehicular VLC applications, where channel conditions are highly dynamic and a rapid reaction is essential. Even though ROCs correlation functions are not impeccable, these codes are adequate to enable numerous nodes to share the same VLC channel. In their case, each user in the network is dispensed with an individual signature code. Next, *L_b_* bits are divided into *Lsc* chips, where *Lsc* represents the length of the ROC. Only ω_c_ chips with a non-zero power level are transmitted, where ω_c_ represents the coding weight, and the placements of ω_c_ non-zero chips appear randomly in *Lsc* chips. Table 1 provides an example of such codes, whereas the correlation between codes is shown in Figure 2. The magnitude of the cross-correlation signal indicates the resemblance between the received signal and the target signal. Although the codes are not orthogonal, the cross-correlation indicates that similarity is substantial in some points. Additionally, when the data bit is one, these code series carry information, whereas a cyclic alteration can be applied in order to modify the original ROC and to generate additional codes. To diminish the effects of interference, the different transmitters were set to have different shifts, enabling improved recovery of broadcast signals, whereas the receiver uses a shift register to perform a matched-filter operation. Nevertheless, as one can see in Figure 2b,c, there are situations in which the cross-correlation between different codes can be very low, which means facile code separation, but also situations in which cross-correlation can have higher values.

### 3.2. Prime Codes

Prime Codes (PCs) are relatively simple codes that benefit from a simple code generation algorithm and simple encoder/decoder design. On these grounds, PCs have been extensively used in non-coherent OCDMA as well as in wavelength hopping OCDMA systems. Nevertheless, PC’s correlation functions are not the best, whereas its auto-correlation signals are often substantial, affecting the reliability of the synchronization for both transmitters and receivers. As a consequence, such codes are unsuitable for asynchronous VLC-CDMA systems, imposing PC improvements. Therefore, modified prime sequence codes have been developed, resulting in Generalized Modified Prime Sequence Codes (GMPSC) and in inverted GMPSC [60]. Thus, GMPSC have improved correlation functions, being this way suitable for MUI cancellation. Table 2 shows the four (ω_c_) main codes and their sub-codes employed in [60].

**Figure 3:**
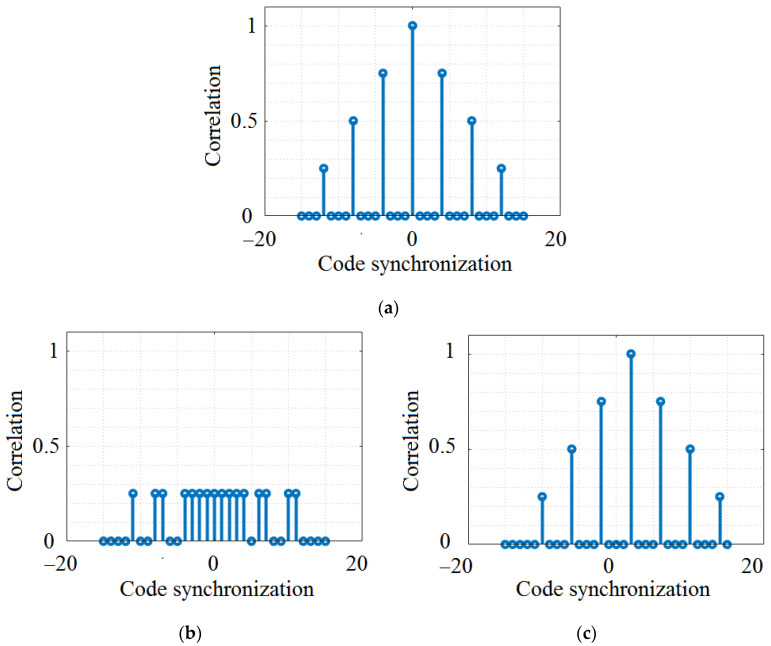
Analysis of PC correlation properties: (**a**) PC3 auto-correlation property; (**b**) example of low cross-correlation between PC3 and PC16; (**c**) example of high cross-correlation between PC3 and PC1.

As one can see, the instigated sub-codes involve splitting the main codes in 4-bit structures and restructuring the position of the frames. Figure 3 illustrates an example of the normalized correlation of the PC. Again, one can see that the code exhibits very good cross-correlation properties when codes from different n_c_ groups are selected (Figure 3b), and less optimal cross-correlation properties if codes from the same n_c_ groups are analyzed (Figure 3c).

### 3.3. Pseudo Noise Codes 

**Figure 4:**
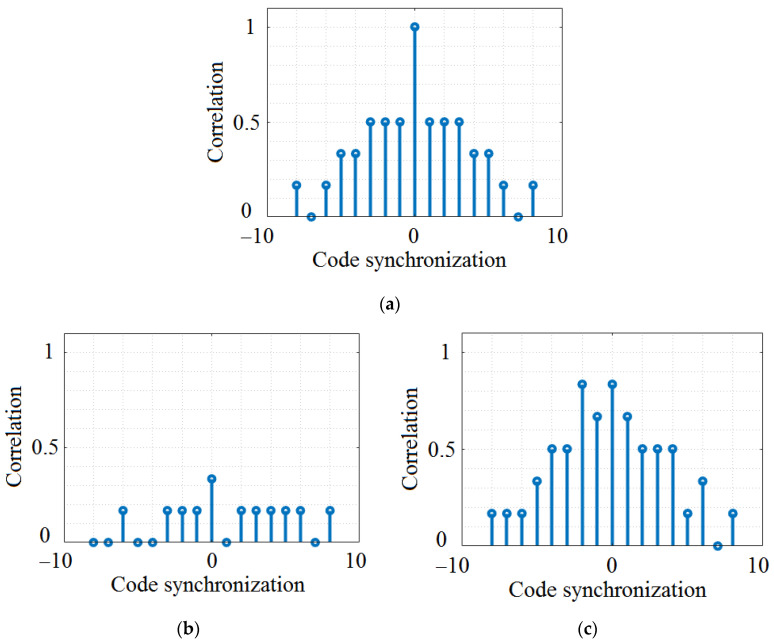
Analysis of PN codes correlation properties: (**a**) PN3 auto-correlation property; (**b**) example of low cross-correlation between PN3 and PN1; (**c**) example of high cross-correlation between PN3 and PN7.

As their name suggests, Pseudo Noise (PN) codes are deterministic binary sequences that, although they appear as channel noise, have a deterministic configuration. PN codes are regularly generated based on feedback shift registers, which are generated using a process that uses a seed value as a start value. The method is deterministic and generates a series of integers that is not statistically arbitrary, but which has a rather high randomness degree, and which are referred to as pseudo-random numbers [61,62]. PN codes are well-suited in OCDMA systems as they can be easily generated and synchronized. Table 3 shows the PN codes that have been considered in this work, whereas Figure 4 provides an example of auto- and cross-correlation. As one can see, the code shows a well-differentiated auto-correlation for the case when the two codes are synchronized. On the other hand, as can be seen in Figure 4, in the best case, the maximum correlation value is lower than 0.35 for auto- and cross-correlation when the signal is not synchronized, whereas in the worst case, maximum cross-correlation can reach a rather high value of 0.83.

### 3.4. Optical Orthogonal Codes (OOCs)

**Figure 5:**
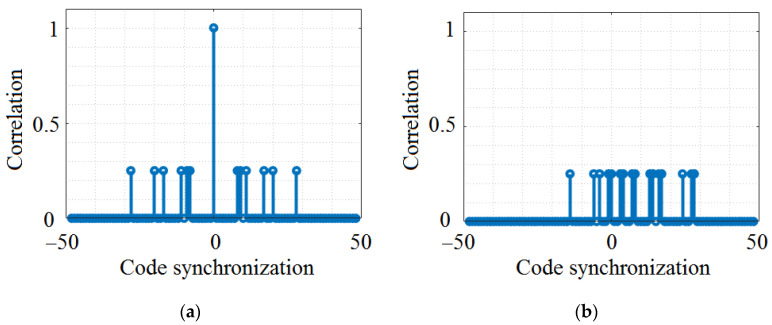
Analysis of OCC correlation properties: (**a**) OOC1 auto-correlation property; (**b**) OOC1 cross-correlation property against other OCC codes, in this case OOC3.

The basic structure of the OOC design was defined in 1989, and is detailed in [63,64]. OOC are designed from 0s, and 1s has a length of *L_c_* and a weight ω_c_, with the imposed condition that they are orthogonal or nearly orthogonal to each other. Auto-correlation and cross-correlation are two of these characteristics. Consequently, OOCs enable a relatively high number of users to transmit data simultaneously over the same optical channel without interfering with each other. OOCs are a type of code that are particularly effective at mitigating interference between users. A main advantage of OOCs is that they can support a large number of users, as the codes are designed to be orthogonal or nearly orthogonal to each other. Nevertheless, the design and implementation of OOCs is more complex than other types of codes previously mentioned. Table 4 presents the description of the codes implemented in this work. As shown in Figure 5a, codes have impulsive behaviors, enabling the differentiation between different codes, whereas the cross-correlation is significantly lower, approaching the zero value. It worth mentioning that in the case of OCC, all codes exhibit similar cross-correlation properties, preventing false code detection.

### 3.5. Comparison between Codes

Figure 2, Figure 3, Figure 4 and Figure 5 graphically present the correlation characteristics of the codes under analysis. As expected, OOCs are the best option in terms of auto-correlation and cross-correlation, having values that do not exceed 0.3 for the case of non-synchronized auto-correlation. Table 5 summarizes the parameters and the correlation properties of the four investigated codes.

One can easily observe that the OOC codes are potentially the best solution because the cross-correlation is always lower than 0.3, while the auto-correlation is 1, which means that codes can be easily distinguished. The ROC codes can be a solution, too, but will probably induce more errors. The PC and PN codes can potentially be performants because of a potential cross-correlation that is quite low, but in some cases, it can be not so easy to distinguish them from a cross-correlation that can be up to 0.8. The results presented in Table 5 also show that when the difference between the length of the code and the number of the code is higher, the maximum cross-correlation is smaller and the different codes are near to orthogonal code.

## 4. Simulation Results and Discussions

After analyzing the performance of the codes from the correlation perspective, in the next section, the effectiveness of each code in a MUI scenario will be evaluated. Table 6 summarizes the simulation setup and the parameters used for the simulations.

### 4.1. Performance Comparison of OCDMA Codes in a Single Interefence Vehicle Scenario

**Figure 6:**
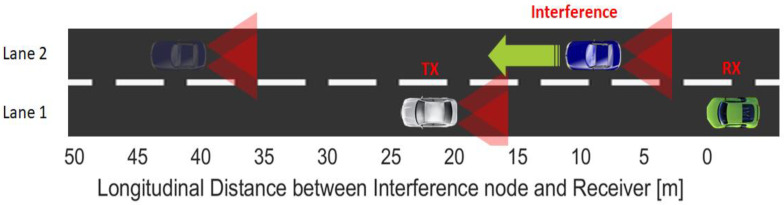
Two lane scenario used to evaluate the performance of the four OCDMA codes.

As illustrated in Figure 6, this simulation scenario envisions an emitter vehicle (TX) and a receiver vehicle (RX) located on a two-lane straight road/highway, and separated by a 20 m distance. An interference vehicle travels in the same direction on the neighboring lane, while broadcasting a random message. The interference vehicle uses the same type of OCDMA protocol as the intended transmitter, but encoded with a dissimilar specific code that has been randomly selected. For these simulations, the received PDR will be determined as the distance between the interference vehicle and the intended receiver increases from 1 to 50 m for each of the four codes. As these simulations are based on the classical VLC channel model described in [65], and also presented in [28], the model is not detailed in this work again.

**Figure 7:**
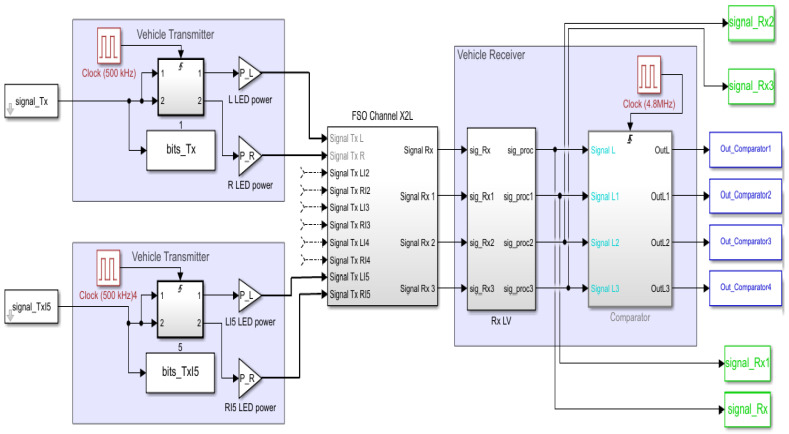
MALAB model used for simulations.

The simulation model illustrated in Figure 7 was run several times for different code arrangements for each group, ROC, PC, PN, and OOC, respectively. For the case of ROC, PC, and PN codes, the simulation scenario also envisions the evaluation of the cases when the two selected sub-codes have maximum and minimum cross-correlations (in accordance with the results provided in Section 3). In the case of OCC, as all codes have similar cross-correlation properties given by the intrinsic orthogonality of the code, such a scenario has not been tested.

**Figure 8:**
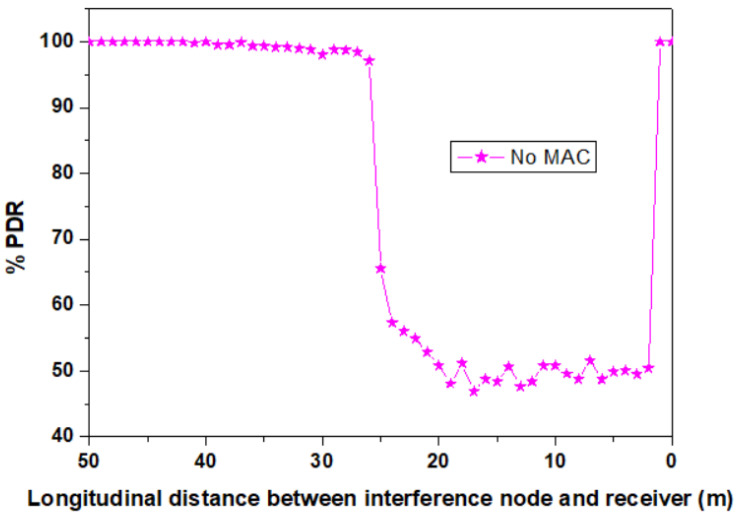
Evaluation of multi-user interference effect when no-MAC solution is used.

**Figure 9:**
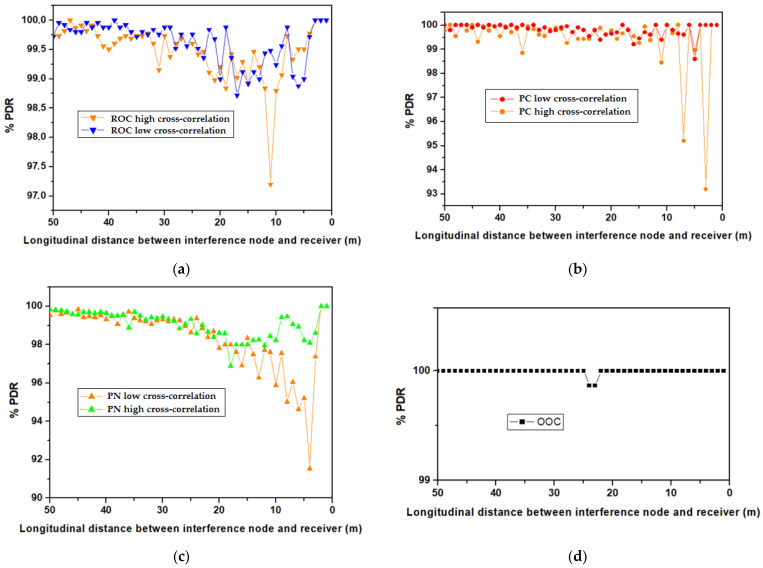
PDR comparison between the four OCDMA codes showing the PDR results for the scenarios when minimum and maximum cross-correlations codes are selected: (**a**) ROC; (**b**) PC; (**c**) PN; (**d**) OOC.

In order to have an idea regarding the effect of MUI in no MAC conditions, the scenario when no MAC is used has been evaluated as well. The simulation results are shown in Figure 8 for the case when no MAC has been used, whereas the results showing the benefits of the four OCDMA codes are presented in Figure 9. As one can see, the lack of MAC use generates a PDR that goes down to around 50–55% for the case when the distance between the interference vehicle and the intended receiver is between 3 and 20 m, with a PDR increasing to 100% when this distance increases above 30 m and the direct LoS with the intended RX receiver is lost. On the other hand, the results shown in Figure 9 confirm the benefits of using the four OCDMA codes. Furthermore, as one can see, the codes are effective even in cases where the selected codes have higher cross-correlation properties. The results reconfirm the superiority of OOC codes in terms of PDR improvement. To highlight the characteristics of each code, the PDR average has been determined and used as a qualitative tool. The simulation results reconfirm the supremacy of OOC codes with a PDR of around 100% for almost the entire service area. This high PDR is justified by the intrinsic orthogonality properties of the OCC, and also by the low correlation value that can be found between the intended transmitter code and the interfering code. Then again, in the case of the non-orthogonal codes, the simulation results reveal that PC codes have the highest PDR means. In their case, the mean value is higher than 99.7% when codes with low cross-correlations are selected. Next, one can find the ROC codes that show the widest performance variation. Lastly, PN codes exhibit slightly lower PDR values, having an average PDR of 98.6. On the other hand, one can see that when codes with higher cross-correlation are examined, the PDR is somehow affected. Although, in this case, the PDR decrease average is not significant, one can still see that at certain points, the PDR can decrease by up to 5–8%. Such PDR drops can be seen in the 3–20 m interval, where the effect of the interferences is higher. Next, after the distance between the interference vehicle and the intended VLC receiver increases above 20 m, their direct LoS is gradually lost, and the effects of the higher cross-correlation between the codes is no longer visible.

### 4.2. Performance Comparison of OCDMA Codes in a Multiple Interefence Vehicles

**Figure 10:**
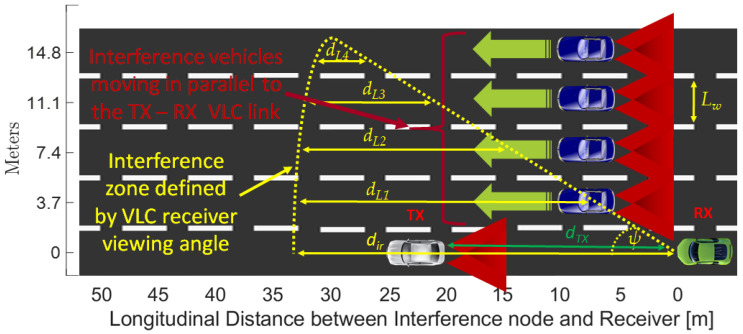
Five lane scenario used to evaluate the performance of the four OCDMA codes.

**Figure 11:**
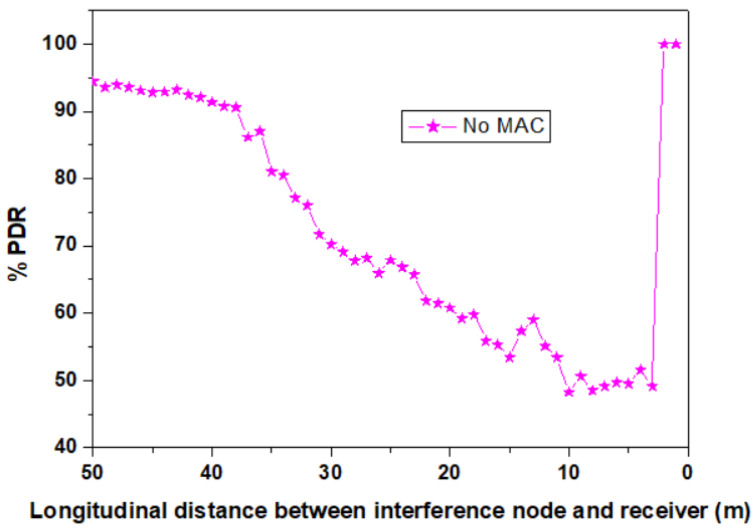
PDR distribution for the five lane scenario in the case when no MAC solution is considered.

After evaluating the effectiveness of the OCDMA codes in limited interfering conditions associated with a two lane road, the next step of the evaluation focuses on an extreme scenario in which four interfering vehicles are considered. The envisioned scenario is illustrated in Figure 10 and it is focused on a five lane strait highway. As this simulation scenario is more complex with respect to the previous one, additional information must be provided. Consequently, it must be remembered that VLC is a technology in which the direct LoS is mandatory. Therefore, in order to be received by the RX vehicle, any node (i.e., intended transmitter or interference vehicles) must be within the receiver’s FOV. In these circumstances, the MUI zone for a given pair of intended transmitter (TX) and receiver (RX) is the circular sector with a radius d_ir_ and center angle ψ, as illustrated in Figure 10. It should also be remembered that the optical power incident on the PD surface is cosine dependent with respect to the incidence angle, meaning that optical signals received from a transmitter located at wider angle with respect to the VLC receiver are less disturbing. Thus, the simulations are carried out on a five-lane straight road, with the receiver (RX) and the intended transmitter (TX) placed on the first lane. The four interfering nodes move at the same time, with a 1-meter step. For each vehicle, the codes are arbitrarily allocated, meaning that the intended TX uses one code, whereas neighboring vehicles use a random sub-code that is different from the code used by the intended TX. For each 1-meter step, the PDR is computed and the final results are established as the average of several passages. As in the previous case, the first simulations considered the case when no MAC is used. The simulation results for this scenario are shown in Figure 11. One can see that without any MAC solutions, a 3 to 38 m interval in which the PDR is below 90% can be observed. Compared to the previous situation, this interval has an extended range that can be explained by the geometry of the four interference vehicles with respect to the position of the intended RX. Thus, in this case, some interference vehicles are within the RX LoS, even for distances above 20 m.

**Figure 12:**
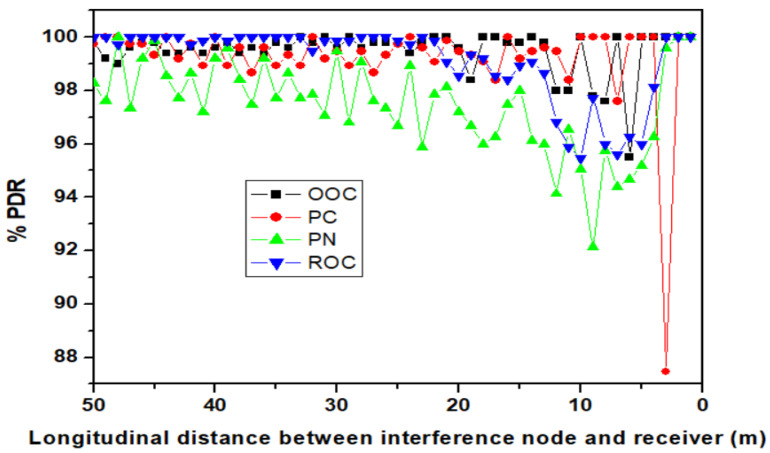
PDR distribution for the five lane scenario in the case when the four optical CDMA solutions are considered.

The simulation results showing the effect of the four OCDMA codes are presented in in Figure 12. Again, the results show a better PDR performance for OOC, but also indicate high robustness to interference for the other codes as well. Thus, as one can see, the four interference vehicles affect the PDR of all the codes, taking the minimum values at 95.5, 87.5, 92.1, and 95.5% for OCC, PC, PN, and ROC, respectively. With one exception, the codes manage to maintain the PDR at values higher than 90% within the entire service area, even under the influence of four interference vehicles, which sometimes are closer to the VLC receiver than the intended TX transmitter. Simulation results also indicate that the lower complexity of PN codes is visible in PDR performance, as this code provides on average a PDR lower by 2% with respect to the other codes.

### 4.3. Comparison of Simulation Time

As a final point, in order to evaluate the employment feasibility of the investigated MAC protocols, the effects of these codes on the introduced latency shall be investigated. In this case, the generated latency is given by the required processing time or in this case by the simulation time required to encode, transmit, and decode a certain frame. In the next evaluation, a 32-byte frame will be considered for each of the four codes. The transmission time is considered to be the period of time from the moment the frame transmission begins and until it ends. Thus, the transmission time will be modified for each OCDMA code in accordance with the dimension of each code and as a function of the coding and decoding process complexity. It should be clarified that the transmission time must not be taken as the propagation delay, which represents the time it takes for the initial bit to travel from transmitter to the receiver, and it depends on the transmission frequency and on the size of the transmitted frame.

For an optical wireless link, the propagation speed is dependent on the communication channel being equivalent to the speed of light, which, in this case, is estimated at 3 × 10^8^ m/s. Thus, as the simulation scenario considered a 20 m distance between the two vehicles, the propagation time can be estimated at 66.79 ns. Consequently, one can define the Packet Delivery Time (*PDT*) or latency as the period of time between the moment when the transmitter begins to send the message and the time when the last bit is decoded by the receiver. In the simulation, the coding and decoding time has been added in order to emphasize the impact of the complexity of the coding/decoding operations for the implemented OCDMA codes, and to evaluate the impact of the code complexity on the overall latency. Therefore, the PDT can be defined as in Equation (1).

(1) PDT=Coding time+Propagation time+Transmission time+Decoding time

**Figure 13:**
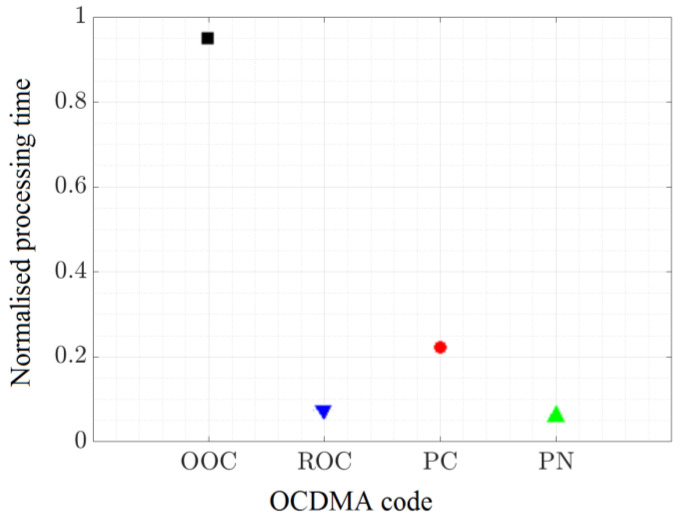
The duration required to simulate the transmission of 32 bytes of data using the different implemented codes: OOC, ROC, PC, and PN.

The results of this evaluation are summarized in Table 7 and in Figure 13, and they show that the performances and the complexity of the OOC codes increase the processing time by five times compared to ROC, PC, and PN codes. The results also emphasize the overall lower simulation time associated with the non-orthogonal codes, with ROC and the PN codes having a significantly shorter simulation time. Once again, it needs to be clarified that these values do not provide the latency of the link, but, rather, the simulation time associated with each of the codes. Therefore, the presented values provide a comparison between the selected code, and, hence, the values should not be taken ad litteram. Although the results were somehow predictable and related to the length of the codes, their quantification remains important, especially for time-critical applications such as communication-based vehicle safety applications. In such applications, the reaction time is vital, and, consequently, latencies below 100 ms and sometimes below 20 ms are imposed [15]. Additionally, as vehicular application messages generally have a length of 208-904 bits, even greater transmission times and latencies are expected.

## 5. Debate on the Results This Work and Future Directions in Optical Code Design for Vehicular Visible Light Communications

### 5.1. Discussion on the Simulation Results and on the Lessons Learned

#### 5.1.1. Debate on the Results and on Their Limitations

Simulation results presented in Section 5.1 and Section 5.2 indicate that OCDMA codes have the potential to enable vehicular VLC systems to be compatible with multi-user application, offering the capability to mitigate the effects of MUI. Thus, these results showed that such codes can provide high PDR even in extreme MUI conditions, as in the case when the interference source is significantly closer to the VLC receiver than the intended transmitter, or in the case when the VLC receiver is exposed to numerous interference sources. According to the results, OCDMA codes can provide a PDR higher than 90%, even in such conditions. Nevertheless, as this work is focused on the effects of MUI, it must be clearly stated that the results only show that the use of different OCDMA codes can mitigate the effects of MUI. Nevertheless, these simulations did not consider the effects of other phenomena that could affect vehicular VLC, such as strong sunlight, unfriendly weather conditions (i.e., snowfall, rain, fog), long-range communication, or transmitter—receiver misalignment conditions. As a matter of fact, these conditions or combination of such factors could further affect the PDR [66]. However, as this work is strictly focused on addressing the MUI effects in vehicular VLC applications and on evaluating four OCDMA codes, such factors have not been considered based on a twofold motivation:

- OCDMA codes are not intended to improve resilience to optical noise or to mitigate the effects of severe weather conditions, nor to improve communication range or to compensate vehicle misalignment; therefore, evaluating them in such conditions is irrelevant; and, furthermore, information concerning techniques and solutions to address such conditions can be found in [8,17,19,20,21,22,23,24,25,26,27].
- As this article is focused on MUI effect analysis, mixing different conditions would not emphasize the cause of a lower PDR; in such circumstances, lower PDR could have been resulted from MUI or from lower SNR generated by, for example, unfriendly weather conditions.

Accordingly, we need to emphasize that cumulation of MUI with additional perturbing factors can lead to a lower PDR.

#### 5.1.2. Debate on the Necessity of a PDR—Latency Trade off and the Importance of Improved Code Allocation Algorithms

The simulation results have shown that each of the four OCDMA codes that have been evaluated is beneficial to mitigating the effect of MUI, and that OCDMA codes significantly improve the performance of vehicular VLC applications. As the results showed, an average PDR higher than 97% can be maintained even in direct exposure to multiple interfering VLC transmitters. Here, it has been shown that codes such as OCC, which are more complex and which have better orthogonality, can provide an average PDR around 99%. On the other hand, simulation results have also shown that in some cases, the complexity of the selected codes can lead to increased data processing times, which, in turn, can affect the communication latency. Moreover, these simulations considered a 256 bit packet size, whereas the payload of a message can climb up to 904 bit or even more [15], and latencies above the imposed 20–100 ms limits could be expected. As in communication-based vehicle safety applications, low latencies are just as important as the high PDR, and compromise solutions that manage to provide simultaneous high PDR and low latencies must be ensured.

Another aspect that needs to be emphasized is related to the cross-correlation of the codes. As shown in Section 3, in some cases, the proposed codes can have very good autocorrelation properties and, also, very low cross-correlation (see the case of OCC in Figure 5). In such cases, high PDR can be expected, as has been confirmed by the simulation results. Nevertheless, in other cases (see the case of PC and PN codes in Figure 3 and Figure 4), the auto-correlation properties are not as good, whereas the cross-correlation properties can differ within the same code. For example, in the case of PC, we can see a low cross-correlation between codes PC when we select codes belonging to different n_c_ groups (see Figure 3b), whereas a significantly higher cross-correlation can be observed when the codes belong to the same n_c_ group (see Figure 3c). Consequently, if two neighboring transmitters randomly select two codes that have good (i.e., low) cross-correlation, higher PDR can be obtained, and vice versa, if codes with high cross-correlation are randomly selected. Based on these considerations, we can say that future work should focus on developing improved algorithms for OCDMA code selection. These algorithms should be able to evaluate the context (e.g., evaluate the VLC channel conditions, the number of lanes, the number of transmitters in the area, and the priority of the data), and based on this evaluation, decide on the type of code to select. For example, pre-crash sensing for cooperative collision mitigation applications require latencies below 20 ms, whereas curve speed warning applications can tolerate latencies up to 1000 ms [15]. Therefore, an algorithm which determines OCDMA selection based on the type of application can ensure a fair tradeoff between PDR and latency.

Consequently, we consider that although very encouraging, the simulation results provided by this article indicate that additional work is required towards the development of improved MUI interference techniques and also towards the development of improved algorithms for MUI management based on environment and context adapting approaches [67,68].

### 5.2. Discussion on the Future Challenges in Optical CDMA Design for Vehicular Visible Light Communication Applications

Optical CDMA codes have the potential to provide real benefits for vehicular VLC applications. Nevertheless, there are still challenges that must be addressed before having vehicular VLC systems ready for deployment in real applications.

An important challenge is related to the need to further improve existing codes and to develop new ones. While addressing this challenge, higher spectral efficiency optical CDMA codes should also be envisioned. As vehicular VLC have limited bandwidth, optical CDMA codes design must focus on the need to maximize spectral efficiency while minimizing the cross-correlation between different codes. Optical CDMA improvement should be also orientated on optimizing code parameters, focusing on optimizing code length, weight, and separation. This issue should be addressed through extensive simulations meant to evaluate the performance of different code parameters in different vehicular VLC scenarios. An additional goal is to improve code detection techniques in order to accomplish reliable code detection in optical noise, mobile, or highly unstable channel conditions. Another major challenge is associated with the need for resilience to mobility-induced channel variabilities. Vehicular VLC systems are exposed to channel variations due to the dynamicity of vehicles, being subject to signal fading and distortion. Optical CDMA codes should be designed to provide resilience to such oscillations in order to provide reliable connections. Some of the upper-mentioned challenges may require advanced signal processing algorithms, which could be enabled based on emerging machine learning, neuronal networks, or artificial intelligence techniques.

As simulations are just one step before having a real product or an operating solution, it is important to advance from simulations to real-life experiments meant to evaluate the performance of optical CDMA codes in realistic vehicular VLC scenarios. This approach is useful to help identify the optimal code parameters for different scenarios, and represents a mandatory condition for technological evolution.

Next, in the context in which standardization is vital for technology development and for its market deployment, optical CDMA codes should be compatible with existing, or, in this specific case, under development communication standards and protocols. This requires coding schemes that can be assimilated with standard communication protocols, or developing new protocols that can take advantage of the unique features of optical CDMA codes. In order to have a widely accepted and widely integrated optical CDMA solution for vehicular VLC applications, it is recommended to have extended collaborations between relevant academic research entities, industry partners, and standardization authorities. Such an approach will help develop practical solutions that can be integrated within existing and under development communication protocols and standards.

In conclusion, further developing optical CDMA codes for vehicular VLC applications involving a multi-disciplinary approach focused on new code design, code parameters optimization, code detection technique enhancement, experimental evaluations in relevant scenarios, and collaborations with industry partners and standardization bodies, standardization, and, in the end, technology deployment.

## 6. Conclusions and Final Debate on the Results and on the Findings of This Work

In the context in which VLC technology has gained a lot of attention and is being considered one of the suitable candidates for inter-vehicle communication applications, this article focused on the issues associated with MUI that could affect the performance of such applications. In its first part, this work has provided a survey focused on MUI effects in vehicular VLC, presenting some of the existing solutions and research directions. Next, the analyses pointed out that due to a relatively lower complexity and high performance, OCDMA codes represent a solution for MUI mitigation. Next, four OCDMA codes have been considered for evaluation: random optical codes, prime codes, pseudo noise codes, and optical orthogonal codes.

The outcomes provided by this article represent a new step toward the practical implementation of VLC technology in automotive applications, providing the future basis for the future implementation in autonomous vehicles. Validated by simulations, the results showed that OCDMA solutions have the potential to provide exceptional performance even in critical situations for VLC applications. Therefore, the simulation results have confirmed that OCDMA solutions enable adequate PDR, even when the intended receiver is within the direct LoS of more than one VLC transmitter, or in the case when several interference vehicles are in the area. Simulation results showed that the performance of OCC codes can exhibit an average PDR higher than 99%. This article also showed that the non-orthogonal codes enable the VLC receiver to maintain a PDR above 90% with only a small effect on the communication latency. Even more, the PC codes analysis showed a stable PDR and more than decent overall performance, considering the relatively reduced complexity with respect to OCC codes.

Future work on this topic will be focused on testing the benefits of the OCDMA codes with hardware VLC prototypes in conditions similar to the ones encountered in real traffic situations.

## Figures and Tables

**Table 1:** Random optical codes considered for implementation: *L_sc_* = 10, ω_c_ = 3, *Users* = 5.

Code	Value
ROC1	0110010000
ROC2	1010000001
ROC3	0001001100
ROC4	0101000001
ROC5	1000100010

**Table 2:** Prime Codes implemented on this work.

Code	n_c_	N_c_	Value
PC1	0	0	1000 1000 1000 1000
PC2		1	0100 0100 0100 0100
PC3		2	0010 0010 0010 0010
PC4		3	0001 0001 0001 0001
PC5	1	0	1000 0100 0010 0001
PC6		1	0100 1000 0001 0010
PC7		2	0010 0001 1000 0100
PC8		3	0001 0010 0100 1000
PC9	2	0	1000 0010 0001 0100
PC10		1	0100 0001 0010 1000
PC11		2	0010 1000 0100 0001
PC12		3	0001 0100 1000 0010
PC13	3	0	1000 0001 0100 0010
PC14		1	0100 0010 1000 0001
PC15		2	0010 0100 0001 1000
PC16		3	0001 1000 0010 0100

**Table 3:** Pseudo-noise codes implemented in this work.

Code	Value
PN1	100 000 100
PN2	001 100 010
PN3	100 111 101
PN4	000 111 001
PN5	001 011 011
PN6	101 100 110
PN7	101 011 111

**Table 4:** Optical orthogonal codes (49, 4, 1, 1) implemented on this work.

Code	Value
OOC1	1100100 0000000 1000000 0000000 0000000 0000000 0000000
OOC2	1010000 1000000 0000000 0000010 0000000 0000000 0000000
OOC3	1000001 0000000 0000100 0000000 0000010 0000000 0000000
OOC4	1000000 0100000 0001000 0000000 1000000 0000000 0000000

**Table 5:** Correlation comparison of OCDMA codes.

Code	*L_SC_*	N_C_	Min Cross-Correlation	Max Cross-Correlation
OOC	49	4	0.25	0.25
PC	16	16	0.25	1
PN	9	7	0.16	0.83
ROC	10	5	0.33	0.66

**Table 6:** Simulation parameters used for OCDMA codes evaluation.

Parameter	Value
PD reference	AFH-206 k
PD active area	7.02 mm^2^
PD responsivity	0.62 A/W
PD capacitance	72 pF/m^2^
VLC receiver FOV (2ψ)	±60°
VLC transmitter half angle	20°
Vehicle width	2 m
VLC trasmitter transmission power	2 Watt
Transmission frequency	500 kHz
Pachet size	256 bits
Number of lanes	2 or 5
Numer of interfering vehicles	1 or 4
Lane width (L_w_)	3.7 m

**Table 7:** Comparison of transmission time of the proposed OCDMA codes.

Code	Transmission Time	Transmission Frequency	Packet Size
OOC	49 ms	500 kHz	256 bits
PC	16 ms	500 kHz	256 bits
ROC	10 ms	500 kHz	256 bits
PN	9 ms	500 kHz	256 bits

## Data Availability

Not applicable.
